# Serum chemerin levels are independently associated with quality of life in colorectal cancer survivors: A pilot study

**DOI:** 10.1371/journal.pone.0176929

**Published:** 2017-05-05

**Authors:** Jee-Yon Lee, Mi-Kyung Lee, Nam-Kyu Kim, Sang-Hui Chu, Duk-Chul Lee, Hye-Sun Lee, Ji-Won Lee, Justin Y. Jeon

**Affiliations:** 1 Department of Family Medicine, Yonsei University, College of Medicine, Seoul, Republic of Korea; 2 Department of Family Medicine, CHA University College of Medicine, CHA Bundang Medical Center, Chaum Life Center, Seoul, Republic of Korea; 3 Department of Sport and Leisure Studies, Sports Medicine Laboratory, Yonsei University, Seoul, Republic of Korea; 4 Department of General Surgery, Yonsei University College of Medicine, Seoul, Republic of Korea; 5 Department of Clinical Nursing Science, Yonsei University, College of Nursing, Nursing Policy Research Institute, Bio-behavioural Research Centre, Seoul, Republic of Korea; 6 Department of Biostatistics, Yonsei University, College of Medicine, Seoul, Republic of Korea; 7 Department of Family Medicine, Gangnam Severance Hospital, Yonsei University College of Medicine, Seoul, Republic of Korea; University Hospital Llandough, UNITED KINGDOM

## Abstract

**Background:**

Colorectal cancer (CRC) survivors are known to experience various symptoms that significantly affect their quality of life (QOL); therefore, it is important to identify clinical markers related with CRC survivor QOL. Here we investigated the relationship between serum chemerin levels, a newly identified proinflammatory adipokine, and QOL in CRC survivors.

**Methods:**

A data of total of 110 CRC survivors were analysed in the study. Serum chemerin levels were measured with an enzyme immunoassay analyser. Functional Assessment of Cancer Therapy (FACT) scores were used as an indicator of QOL in CRC survivors.

**Results:**

Weak but not negligible relationships were observed between serum chemerin levels and FACT-General (G) (r = -0.22, p<0.02), FACT-Colorectal cancer (C) (r = -0.23, p<0.02) and FACT-Fatigue (F) scores (r = -0.27, p<0.01) after adjusting for confounding factors. Both stepwise and enter method multiple linear regression analyses confirmed that serum chemerin levels were independently associated with FACT-G (stepwise: β = -0.15, p<0.01; enter: β = -0.12, p = 0.02), FACT-C (stepwise: β = -0.19, p<0.01; enter; β = -0.14, p = 0.02) and FACT-F scores (stepwise: β = -0.23, p<0.01; enter: β = -0.20, p<0.01).

**Conclusions:**

Our results demonstrate a weak inverse relationship between serum chemerin and CRC survivor QOL. Although it is impossible to determine causality, our findings suggest that serum chemerin levels may have a significant association with CRC survivor QOL. Further prospective studies are required to confirm the clinical significance of our pilot study.

## Introduction

Colorectal cancer (CRC) is one of the most common cancers worldwide [1]. The survival rate of CRC patients has steadily improved in large part due to the increased rate of early detection and more effective treatments [2]. CRC survivors are known to suffer from various symptoms, including chronic bowel irritability and cancer-related fatigue that significantly affect their quality of life (QOL) [3]. Therefore, long-term care that improves QOL in CRC survivors is considered important. Furthermore, the identification of clinical markers and biomarkers that allow clinicians to predict the QOL of CRC survivors are needed. Although the precise mechanism is unknown, immune system alterations and increased expression of pro-inflammatory cytokines are considered to mediate QOL decreases. In previous studies, increased cytokine levels in cancer patients were significantly associated with non-specific chronic symptoms, such as fatigue, cognitive changes, and depressed mood [4, 5].

Chemerin is a newly identified adipokine secreted by adipose tissue [6]. It is known to be associated with carcinogenesis and several age-related metabolic disorders, including obesity, metabolic syndrome, and insulin resistance (IR) [7, 8]. Although the precise role of chemerin has not been fully elucidated, it is known to modulate immune system function and enhance the pro-inflammatory process by stimulating the chemotaxis of dendritic cells and macrophages [9–11].

Because decreased QOL in CRC is considered to be associated with immune system alterations and chronic systemic inflammation, it is possible that the pro-inflammatory adipokine chemerin may be associated with QOL in CRC survivors. In the present study, we investigated the relationship between QOL as measured by Functional Assessment of Cancer Therapy (FACT) scores and serum chemerin levels in 110 Korean CRC survivors.

## Materials and methods

### Ethics statement

All subjects participated in the study voluntarily, and written informed consent was obtained from each participant. The study complied with the Declaration of Helsinki, and the Institutional Review Board of Severance Hospital approved this study.

### Study participants

This study was performed as part of a clinical study designed to investigate factors related to the heath of Korean CRC survivors. This study sample included 123 CRC survivors diagnosed with stage 2 or 3 CRC between January 2011 and December 2013. They were all older than 20 years old and had completed all standard treatments between 6 week and 1 year before study enrolment. The performance status of the participants was evaluated on the basis of Eastern Cooperative Oncology Group performance scores, with total scores <1 indicating good performance. The study population was recruited by advertisement at the Outpatient Clinic of the Department of General Surgery in Severance Hospital.

We excluded participants with histories of cancer in other organs and colostomies. Participants with a history of chronic diseases, including coronary artery occlusive disease, stroke, chronic liver disease, or renal disease were not included. Patients with abnormal liver function or kidney function were also excluded. Abnormal liver function was defined by serum aspartate aminotransferase (AST) or alanine aminotransferase (ALT) concentrations >100 IU/L. Abnormal kidney function was defined by serum creatinine concentrations >1.7 mg/dL. No participants had physical or mental disabilities. Subjects who participated in vigorous exercise (defined as high intensity of physical exercise or physical work performed >200min/week) were also excluded. In addition, we excluded participants who were missing data for chemerin levels and FACT scores. A total of 110 participants were included in the final analysis. Among these 110 participants, 66 participants further participated in a randomized controlled trial to assess the effects of probiotics (clinical trial number: KCT0001053). We performed our analysis using baseline measurement data from 110 participants. (S1 Fig)

### Measurements

#### Health-related questionnaires

All subjects completed a questionnaire about lifestyle factors, including exercise, cigarette smoking, alcohol consumption, marital status, and underlying medical conditions. Smoking was defined as a current smoking habit, and alcohol consumption was categorized into never drinking, drinking less than once a week, or drinking alcohol once a week or more. Participants also completed a questionnaire about CRC, including tumour location (sigmoid, ascending colon, transverse colon, descending colon, or rectum), cancer stage (II or III), treatment modality (surgery, chemotherapy, radiotherapy, or combined therapy) and intervals after treatment completion (day).

#### Assessment of cancer-related QOL

All subjects completed the questionnaires about cancer-related QOL assessed by version 4 of the FACT Measurement System [12]. Among the FACT questionnaires, FACT-General (FACT-G), Total FACT-cancer-related (FACT-C), and Total FACT-fatigue (FACT-F) scales were chosen in this study. A five-point Likert self-report scale with scores ranging from 0 to 4 was used, with higher scores indicating better conditions.

The FACT-G is a 27-item questionnaire divided into 4 subcategories: physical well-being (PWB), social well-being (SWB), emotional well-being (EWB), and functional well-being (FWB). The range of possible scores is 0–108.

The FACT-C is a 9-item questionnaire about CRC-related QOL issues, with a range of possible scores of 0–28. The total FACT-C total scores were calculated by summation of PWV, SWB, EWB, FWB, and Fact-C scores (score ranges 0–136).

The FACT-F is a 13-item fatigue subscale. The range of possible scores is 0–52. The total FACT-F total scores were calculated by summation of the PWV, SWB, EWB, FWB, and Fact-F scores (Score ranges 0–160).

#### Anthropometric measurements

Anthropometric measurements were taken by a single well-trained examiner. Blood pressure (BP) was measured in the sitting position after a 10-minute resting period. Body mass index (BMI) was calculated as weight divided by height squared. Waist circumference was measured at the umbilicus while the subject was standing. Bioelectrical impedance analysis was used to estimate body fat percentage and lean body mass using the InBody U20 (Biospace, Seoul, Korea).

Blood samples were collected after at least an 8-hour overnight fasting period. White blood cell counts, fasting glucose, total cholesterol, high-density lipoprotein (HDL) cholesterol, triglyceride, high sensitive C-reactive protein (hs-CRP) and gamma-glutamyl-transpeptidase (GGT) concentrations were measured using an ADVIA 1650 chemistry system (Siemens Medical Solution, Tarrytown, NY, USA). Fasting insulin was determined by electrochemiluminescence immunoassay using an Elecsys 2010 (Roche, Indianapolis, IN, USA). IR was estimated by the homeostasis model assessment of insulin resistance (HOMA-IR) index: (insulin [μIU/mL] × fasting blood glucose [mg/dL]/18)/22.5. Serum chemerin levels were measured with an enzyme immunoassay kit (Mesdia, Seoul, Korea), and the inter- and intra-assay variabilities were 11.3±6.0% and 8.4±3.7%, respectively.

### Statistical analysis

Normally distributed data are expressed as means±standard deviations (SD), and non-normally distributed data are expressed as medians and interquartile ranges. Non-normally distributed data were logarithmically transformed to reduce the skewness of the distribution.

Pearson correlation analysis was performed to evaluate relationships between FACT scores and other clinical variables. To assess the association between FACT scores and serum chemerin levels, Pearson’s partial correlation analyses were performed to determine the correlations between serum chemerin levels and FACT-C, FACT-F and FACT-G scores after adjusting for other variables with p<0.10 from simple Pearson correlation analyses and clinically important variables, including age, sex, smoking and alcohol consumption. To avoid multicollinearity, if there was significant correlation (r>0.7) between two variables, only one variable was selected and entered into the model.

Multiple linear regression analyses with stepwise and enter methods were used to identify factors contributing to FACT scores. For this analysis, the same variables adjusted in the Pearson’s partial correlation analyses were entered. For the comparison of r^2^ between the full model and reduced model (null model) without chemerin, an F-test was performed.

We performed all statistical analysis using the Statistical Package for the Social Sciences, version 18.0 (SPSS Inc., Chicago, IL, USA). Statistical significance was defined as p<0.05.

## Results

Table 1 shows the clinical characteristics of the subjects. The mean age of participants was 56.29±9.27 years, and the mean chemerin level was 104.69±14.31 ng/mL. QOL scores related with general symptoms (FACT-G scores), CRC-related symptoms (FACT-C scores), and fatigue-related symptoms (FACT-F scores) were 80.57±13.68, 100.89±16.53, and 122.40±18.40, respectively.

**Table 1:** Subjects’ clinical characteristics (n = 110).

Variables	Mean±SD or Median (25^th^–75^th^ percentile)
Age (years)	56.29±9.27
Gender (n, %)	
Male	55 (50.0)
Female	55 (50.0)
Marriage (n, %)	92 (82.9)
Smoking status (n, %)	
None	52 (47.3)
Current	24 (21.8)
Past	34 (30.9)
Alcohol consumption (n, %)	
Never	87 (79.1)
<1/week	14 (12.7)
>1/week	9 (8.2)
Cancer location (n, %)	
Colon	
Sigmoid	45 (40.9)
Ascending	21 (19.1)
Transverse	6 (5.5)
Descending	5 (4.5)
Rectum	33 (30.0)
Cancer stage (n, %)	
II	54 (49.1)
III	56 (50.9)
Treatment (n, %)	
Surgery + Chemotherapy	101 (91.8)
Surgery + Chemo-radiotherapy	9 (8.2)
Intervals after treatment completion (day)	257.00 (113.00–418.00)
Adiposity index	
BMI (kg/m^2)^	23.31±3.06
Waist (cm)	81.90±9.10
Lean body mass (cm^2^)^#^	25.40 (20.50–29.00)
Fat (%)	27.33±7.85
BP (mmHg)	
Systolic	130.22±13.66
Diastolic	78.72±11.20
Fasting glucose (mg/dL)^#^	90.00(85.00–98.00)
Fasting insulin (μIU/mL)^#^	5.80 (3.47–8.63)
HOMA-IR^#^	1.31 (0.80–2.03)
Lipid profile (mg/dL)	
Total cholesterol	191.13±35.17
Triglyceride^#^	100.5 (71.00–150.00)
HDL-cholesterol^#^	53.55 (46.50–62.20)
WBC count^#^	4570 (3800–5640)
GGT^#^	22.00(14.00–37.00)
Chemerin	104.69±14.31
FACT-G score	80.57±13.68
Total FACT-C score	100.89±16.53
Total FACT-F score	122.40±18.40

^#^ Non-normally distributed data were logarithmically transformed to reduce the skewness of the distribution.

Abbreviation: SD; standard deviation, BMI; body mass index, BP; blood pressure, HDL-cholesterol; high density lipoprotein cholesterol, WBC count; white blood cell count, Hs-CRP; high sensitive c-reactive protein, GGT; gamma glutamyl transpeptidase, FACT; Functional Assessment of Cancer Therapy.

**Fig 1:**
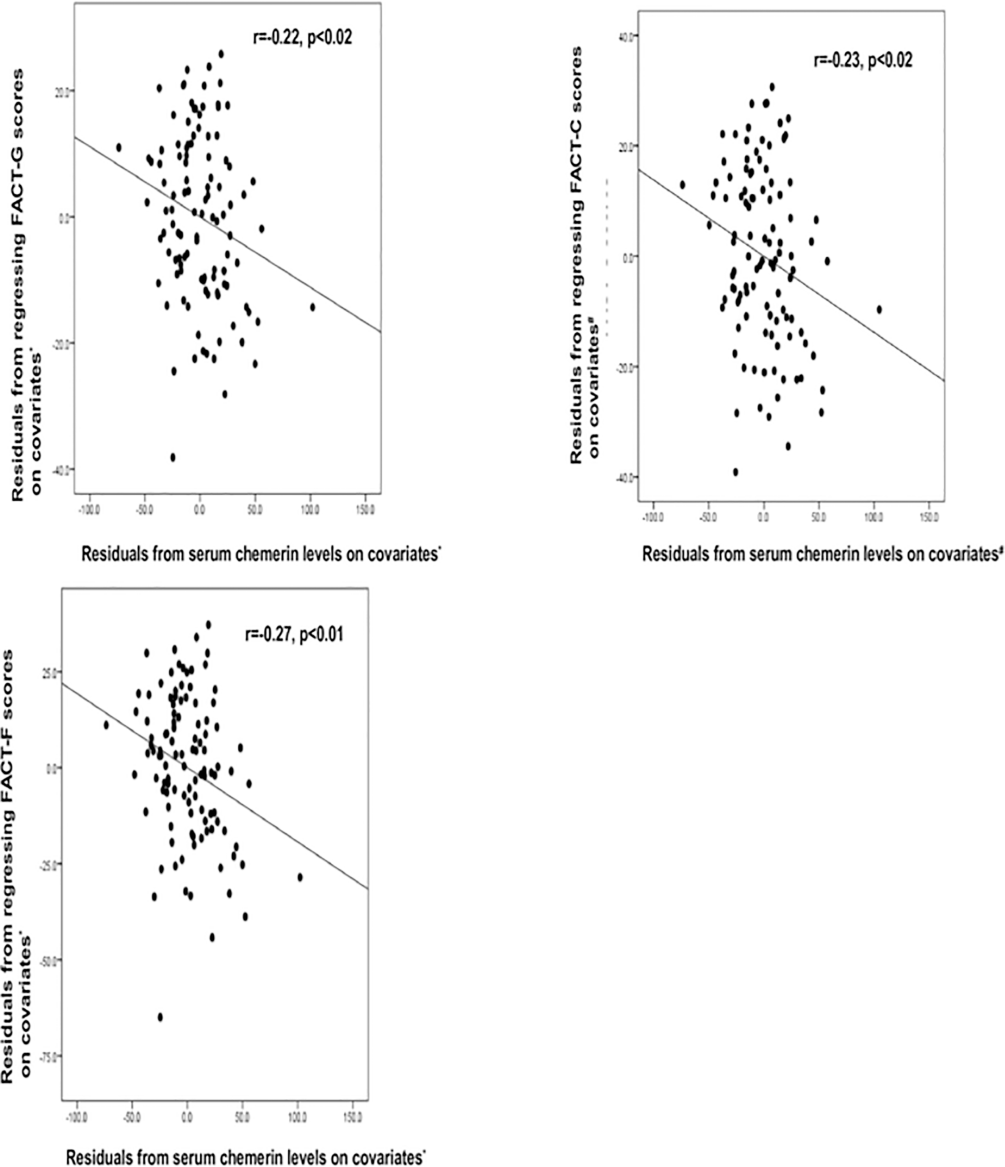
Relationships between serum chemerin level and FACT scores (FACT-G, FACT-C, and FACT-F). r: Pearson’s partial correlation coefficient (r = 0, no linear relationship; r = 1 or -1, perfect linear relationship). X-axis values are based on calculated residuals from regressing serum chemerin levels on covariates* including age, sex, smoking, alcohol, fat %, insulin, and triglycerides (a, c) and covariates^#^ including age, sex, smoking, alcohol, fat %, lean body mass and triglycerides (b). Y-axis values are based on calculated residuals from regressing FACT scores on covariates* including age, sex, smoking, alcohol, fat %, insulin, and triglycerides (a, c) and covariates^#^ including age, sex, smoking, alcohol, fat %, lean body mass, and triglycerides (b).

In correlation analyses, FACT-G and FACT-C scores were negatively related with age (r = -0.19, p = 0.04; r = -0.20, p = 0.04), fat percent (r = -0.17, p = 0.04; r = -0.17, p = 0.04), triglyceride (r = -0.22, p = 0.02; r = -0.22, p = 0.02), high-sensitivity C-reactive protein (hsCRP; r = -0.22, p = 0.02; r = -0.23, p = 0.02) and chemerin levels (r = -0.29, p<0.01; r = -0.29, p<0.01). In addition, FACT-F scores were negatively correlated with triglyceride (r = -0.22, p = 0.02) and chemerin levels (r = -0.31, p<0.01) (Table 2). There was also a significant correlation between chemerin and hs-CRP levels (r = 0.48, p<0.01). The weak but significant correlations between serum chemerin levels and FACT-G and FACT-F scores were maintained after adjustments were made for age, sex, fat percent, smoking, alcohol use, insulin, and triglyceride (r = -0.22, p<0.02 for FACT-G scores; r = -0.27, p<0.01 for FACT-F scores). A weak correlation between serum chemerin level and FACT-C scores persisted after adjusting for age, sex, fat percent, smoking, alcohol use, lean body mass, and triglyceride (r = -0.23, p<0.02) (Fig 1).

**Table 2:** Correlations between cancer-related QOL scores and other clinical parameters.

Variables	FACT scores					
	FACT-G		Total FACT-C		Total FACT-F	
	r	p-value	r	p-value	r	p-value
Age (years)	-0.19	0.04	-0.20	0.04	-0.17	0.08
Adiposity index						
BMI (kg/m^2^)	-0.98	0.30	-0.07	0.47	-0.10	0.32
Waist (cm)	-0.14	0.12	-0.13	0.18	-0.15	0.11
Lean body mass (cm^2^)	0.14	0.16	0.19	0.05	0.12	0.21
Fat (%)	-0.17	0.04	-0.17	0.04	-0.17	0.07
BP (mmHg)						
Systolic	0.03	0.74	0.04	0.65	0.06	0.54
Diastolic	0.01	0.92	0.02	0.81	0.05	0.64
Fasting glucose (mg/dL)	0.07	0.49	0.06	0.54	0.11	0.23
Fasting insulin (μIU/mL)^#^	-0.17	0.06	-0.14	0.14	-0.16	0.09
HOMA-IR^#^	-0.14	0.14	-0.12	0.22	-0.12	0.22
Lipid profile (mg/dL)						
Total cholesterol^#^	-0.06	0.55	-0.04	0.71	-0.03	0.77
Triglyceride^#^	-0.22	0.02	-0.22	0.02	-0.22	0.02
HDL-cholesterol^#^	0.11	0.24	0.12	0.20	0.13	0.17
WBC count	0.05	0.61	0.03	0.74	0.04	0.71
Hs-CRP	-0.22	0.02	-0.23	0.02	-0.23	0.01
GGT	-0.08	0.39	-0.02	0.84	-0.09	0.35
Chemerin	-0.29	<0.01	-0.29	<0.01	-0.31	<0.01
Intervals after treatment completion (day)	0.02	0.83	0.03	0.75	0.04	0.69

Coefficient r and p values were calculated by Pearson’s correlation test

^#^ Non-normally distributed data were logarithmically transformed to reduce the skewness of the distribution.

Abbreviation: BMI; body mass index, BP; blood pressure, HDL-cholesterol; high density lipoprotein cholesterol, WBC count; white blood cell count, Hs-CRP; high sensitive c-reactive protein, GGT; gamma glutamyl transpeptidase

Finally, independent associations of QOL scores with serum chemerin levels were assessed in multivariate-adjusted models by multiple regression analyses with the enter method. Chemerin was identified as a significant independent variable associated with FACT-G (β = -0.12, p = 0.02), FACT-C (β = -0.14, p = 0.02), and FACT-F scores (β = -0.20, p<0.01) after adjustments for age, gender, smoking, alcohol use, fat percent, triglycerides, and insulin for FACT-G, FACT-F and adjustments for age, gender, smoking, alcohol, fat percent, lean body mass, and triglycerides for FACT-C scores. Furthermore, r^2^ increased significantly when comparing between the null and full models (chemerin added) in analyses (FACT-G scores; 0.12–0.16, p = 0.02, FACT-C scores; 0.13–0.17, p = 0.02, FACT-F scores; 0.12 to 0.18, p<0.01). These associations remained significant in stepwise multiple regression analyses (β = -0.15, p<0.01 for FACT-G, β = -0.19, p<0.01 for FACT-C and β = -0.23, p<0.01 for FACT-F scores) after adjustments for age, sex, BMI, alcohol consumption, smoking, marriage, cancer stage, treatment modality, cancer location, intervals after treatment completion, lean body mass, systolic BP, total cholesterol, HDL-cholesterol, triglycerides, fasting glucose, insulin, gamma glutamyl transferase (GGT), and hs-CRP (Table 3).

**Table 3:** Enter method multiple linear regression analyses of serum chemerin, other clinical variables, and FACT scores.

	Enter method						Stepwise method					
	FACT-G			Total FACT-C	Total FACT-F		FACT-G		Total FACT-C		Total FACT-F	
	β (SE)	*p*-value	β (SE)	*p*-value	β (SE)	*p*-value	β (SE)	*p*-value	β (SE)	*p*-value	β (SE)	*p*-value
Age	-0.20(0.16)	0.21	-0.26(0.20)	0.20	-0.16(0.22)	0.47						
Sex	-4.96(3.74)	0.19	-7.32(6.94)	0.29	-6.05(5.15)	0.24						
Smoking	-1.13(3.59)	0.75	-0.84(4.32)	0.85	-3.58(4.95)	0.47						
Alcohol	2.45(3.42)	0.48	2.89(4.10)	0.48	6.44(4.71)	0.18						
Fat (%)	-0.08(0.26)	0.75	-0.06(0.29)	0.84	-0.05(0.35)	0.89						
Lean body mass (kg)			2.92(4.35)	0.84								
Triglyceride (mg/dL)	-2.21(3.18)	0.49	-2.91(3.52)	0.41	-2.23(4.39)	0.61						
Insulin (mU/ml)	-1.53(2.83)	0.59			-1.42(3.89)	0.72						
Chemerin (ng/mL)	-0.12(0.05)	0.02	-0.14(0.06)	0.02	-0.20(0.07)	<0.01	-0.15(0.05)	<0.01	-0.19(0.06)	<0.01	-0.23(0.07)	<0.01

Variables included in the stepwise model for FACT-C scores were age, sex, BMI, alcohol consumption, smoking, marriage, cancer stage, treatment modality, cancer location, intervals after treatment completion, lean body mass, systolic BP, total cholesterol, HDL-cholesterol, triglycerides, fasting glucose, insulin, GGT, hs-CRP, and chemerin levels.

r^2^ = 0.29 for FACT-G scores, r^2^ = 0.31 for FACT-C scores, r^2^ = 0.31 for FACT-F scores in the stepwise mode

## Discussion

Our cross-sectional pilot study showed a weak but significant inverse relationship between CRC-related QOL and serum chemerin levels in 111 cancer survivors. These relationships remained significant after adjustment for other confounding factors that affect cancer survivor QOL.

Given the increasing number of CRC survivors and their many reported health concerns [3], improving the QOL of CRC survivors is considered a very important issue. However, it is very difficult to predict the QOL of each cancer survivor because of the lack of valuable biological predictive markers. Because immune system alterations and increased chronic inflammation is known to affect CRC-related symptoms, including fatigue, loss of appetite and poor performance [13–15], pro-inflammatory cytokines have long been considered as viable marker candidates for cancer-related symptoms. However, the modulatory role of adipokines on inflammatory cytokines and immunologic responses and their effects on QOL remain unknown. To our knowledge, this is the first study to investigate the relationship between CRC-related QOL and serum chemerin levels.

Although the precise mechanism of the relationship between CRC-related QOL and chemerin is still unknown, we suggest the following possible mechanism. Chemerin is an agonist of the orphan G protein-coupled receptor chemokine-like receptor 1(CMKLR1, ChemR23), which is abundantly expressed on antigen-presenting cells (APCs), including plasma dendritic cells, natural killer cells, and macrophages [9–11, 16]. Chemerin induces the initiation of the innate immune response and inflammatory processes by attracting these APCs [16]. Recent studies have demonstrated a positive association between chemerin and several pro-inflammatory cytokines, including interleukin 6 (IL-6), IL-8, and tumour necrosis factor (TNF)-alpha, which is associated with cytotoxic cell-mediated immunity [17, 18]. These cytokines are known to modulate the psycho-neuroendocrine system that induces the chronic non-specific behaviour symptoms termed ‘sickness behaviour’ [19]. Therefore, the pro-inflammatory and immune-modulating properties of chemerin may influence CRC survivor QOL, including fatigue and general symptoms.

Interestingly, we found that chemerin levels were significantly associated with CRC-related QOL as well as general QOL and fatigue-related QOL symptoms. The CRC-related QOL questionnaires are mainly about bowel symptoms including bloating, loose stool, and abdominal pain. Although various factors, including surgical bowel resection [20] and alteration of gut microbial environment [21] collectively contribute to the development of chronic bowel symptoms in CRC survivors, it is hard to predict their occurrence and prognosis. Chemerin is known to be elevated in the inflammatory bowel diseases, including ulcerative colitis and Crohn’s disease [22]. Although is mainly released from adipose tissue [23], it is also synthesized by foetal intestinal epithelial cells [24] and is considered to promote inflammatory process, as well as disturb immune response that result in the chronic inflammatory bowel disease [22, 25]. Therefore the pro-inflammatory and immune-modulating properties of chemerin may also contribute to both the development of chronic bowel symptoms and decreases in general and fatigue-related QOL in CRC survivors.

In addition, we suggest possible non-inflammatory roles of chemerin on QOL in CRC survivors. We observed a significant correlation between chemerin and hs-CRP levels. (r = 0.48, p<0.01) However, in a stepwise multivariate linear analysis, chemerin was independently associated with FACT-G, FACT-C, and FACT-F scores even after adjusting for hs-CRP level. This suggests that chemerin may have an independent role in the decline of quality of life in cancer survivors apart from its role in mediating inflammatory markers. Previously, Dranse et al.[26] have been reported higher proportions of Akkermansia and Prevotella bacteria in a wild type mouse compared with a chemerin knock out mouse. Both these bacteria are known to be increased in the gut of patients with irritable bowel syndrome [27, 28]. Additionally, previous studies also have demonstrated the antimicrobial activity of chemerin[29, 30]. These results suggest a possible role of chemerin in themodulation of the gut microbial community. Alterations of the gut bacterial community have been reported after bowel resection surgery in CRC patients[21] and are known to be related to various bowel symptoms that affect the quality of life in patients[3, 31]. Therefore, although we could not elucidate the precise mechanism underlying the role of chemerin in dysbiosis in the gut bacterial community, chemerin may be associated with CRC related QOL due to its modulatory effect on gut bacterial communities. Further studies that investigate the relationship among the serum chemerin, distribution in the gutbacterial community and quality of life in CRC survivors should be performed in the future.

Despite compelling results, our study has several limitations. First, the cross-sectional study design did not allow us to determine causality. Second the small sample size from a single hospital does not allow for generalization of the data to a larger population. Third, the p values of association between serum chemerin levels and FACT scores were <0.05, the r and r^2^ values were relatively low. However based on the previous studies [32,33], the weak and low-significance relationships found in our pilot study suggest the necessity of performing further large-scale studies to determine the precise relationship between chemerin and quality of life in CRC survivors. Fourth, we cannot exclude the possibility that the relationship between chemerin and QOL in CRC survivors was mediated by unknown confounding factors. Additionally, we did not measure other inflammatory markers except hs-CRP and chemerin levels. Therefore, we could not compare chemerin’s association with FACT measures with those of other inflammatory markers. However, when we compared the effect sizes for chemerin and FACT scores with the results of previous studies that investigated relationships between quality of life scores and various inflammatory markers including interleukin (IL)-6, IL-1-b, IL-2, neoterin, and TNF-alpha, the simple correlation coefficients between chemerin and FACT scores (r = -0.29 to -0.31) were similar to the simple correlation coefficients between other inflammatory markers and cancer-related QOL scores (e.g., IL-6 and FACT-F, r = -0.28; TNF-a and Global QOL, r = 0.31; IL-1b and Fatigue VAS, r = 0.30; IL2 and Global QOL r = -0.27; neopterin and fatigue levels on the LASA scales, r = 0.28)[13, 34–36]. Further studies that investigate the association between various inflammatory markers and QOL in cancer survivors should be performed to find the most valuable candidate for predicting cancer survivor QOL.

In conclusion, serum chemerin levels were weakly but independently associated with QOL in Korean CRC survivors. Although it is impossible to determine causality and to assume clinical consequences based on these weak relationships, we suggest the possibility that chemerin may be considered a candidate biomarker related to QOL in CRC survivors. Further prospective and experimental studies are needed to clarify the clinical significance of our findings.

## Supporting information

S1 Fig Study diagram. (TIFF)
